# Suspected Intestinal Tuberculosis Might Be Crohn's Disease

**DOI:** 10.1155/2010/695461

**Published:** 2010-05-17

**Authors:** Semiu Eniola Folaranmi, Gautam Mehta, Debasis Datta, Greg Holdstock

**Affiliations:** ^1^Department of Paediatric Surgery, Royal Manchester Children's Hospital, Manchester M13 9WL, UK; ^2^Department of Gastroenterology, Institute of Hepatology, University College London, London WC1E 6HX, UK; ^3^Department of Gastroenterology, The Hillingdon Hospital, Middlesex UB8 3NN, UK

## Abstract

In this case report we provide evidence that supports a link between mycobacteria and Crohn's disease. The patient in question, KG, presented on three separate occasions over a ten years period with features suggestive of intestinal tuberculosis. He was treated successfully on each occasion with antimycobacterial drugs. When he presented a fourth time with the same symptoms, he was diagnosed with Crohn's disease based on findings from endoscopy, histology and CT. Subsequently he was treated with a course of steroids and made a full recovery. This case adds weight to the theory that mycobateria has an aetiological role in Crohn's disease.

## 1. Introduction

Crohn's disease (CD) is an inflammatory disorder of the gastrointestinal tract characterised by transmural inflammation. The cause of Crohn's disease remains unknown; however, it is increasingly recognised that mucosal inflammation results from a dysfunctional innate immune response in susceptible individuals. Enteric microorganisms are thought to play a pivotal role in the initiation and maintenance of inflammation, although a specific infectious agent remains elusive. The similarities between Crohn's disease and Johne's disease, a chronic enteritis affecting cattle and other ruminants caused by Mycobacterium avium paratuberculosis (MAP) [1], have led observers to suggest a link between Crohn's disease and mycobacteria.

There is microbiological and serological evidence of an association of MAP with CD. MAP has been cultured in 50% of blood samples of patients with CD, as compared with 20% of patients with ulcerative colitis and 0% of controls [2]. MAP has also been cultured from mucosal biopsies from a greater proportion of CD patients (42%) than from controls (9%) [3]. MAP antibodies have also been found with greater frequency in CD than in control [4]. However, the lack of efficacy of antituberculous therapy in CD has limited any suggestions of causality. In this report we present a case of CD treated with antituberculous therapy, and we review the evidence for a causal role of MAP in CD.

## 2. Case Presentation

KG, a 42-year-old Indian male, presented in 1995 with abdominal pain exacerbated by eating, anorexia, and weight loss. He had travelled to India 5 years previously, and he was known to have beta thalassaemia trait. There was no history of fevers or diarrhoea. He remained afebrile during his inpatient stay. Gastroscopy, colonoscopy, small bowel meal, and barium enema were normal. Early morning urine samples, and Mantoux test, did not demonstrate evidence of tuberculosis. Furthermore, bone marrow biopsy showed leukopenia and thrombocytosis, with no organisms present on culture. His initial blood tests confirmed a thrombocytosis with a platelet count of 645 × 10^9^/L. His haemoglobin was 12.0 g/dL, and his ESR was elevated at 64 mm/hr.

In our experience, the diagnosis of tuberculous enteritis frequently remains challenging despite the investigations described above. Several authorities recommend initiating antituberculous therapy if there is a strong clinical suspicion of tuberculosis despite nondiagnostic histological or bacteriological studies [5]. Whilst laparoscopy and mesenteric sampling have a higher sensitivity, these patients frequently respond rapidly to medical therapy and, thus, laparoscopy is usually considered if improvement is not seen after two weeks. In this case, due to the patient's marked systemic inflammation, Indian origin, and lack of evidence for alternative diagnoses such as lymphoma or inflammatory bowel disease, he was commenced on empirical antituberculous treatment with rifampicin, isoniazid, ethambutol, and pyrazinamide. He gained weight rapidly, and his blood tests returned to normal. He received eight and a half months of treatment.

He relapsed the following year with abdominal pain, vomiting, and fever. CT of the abdomen demonstrated an abnormal terminal ileum and caecum, suggestive of tuberculosis, inflammatory bowel disease, or lymphoma. A small bowel meal confirmed an irregular thickening in the terminal ileum. However bone marrow aspirate and peripheral cultures proved unremarkable, with no evidence of mycobacteria. A diagnostic laparoscopy was recommended, but the patient declined this procedure. A further course of antituberculous therapy with the same regimen again provided a dramatic therapeutic response. He received a year of treatment. 

**Figure 1:**
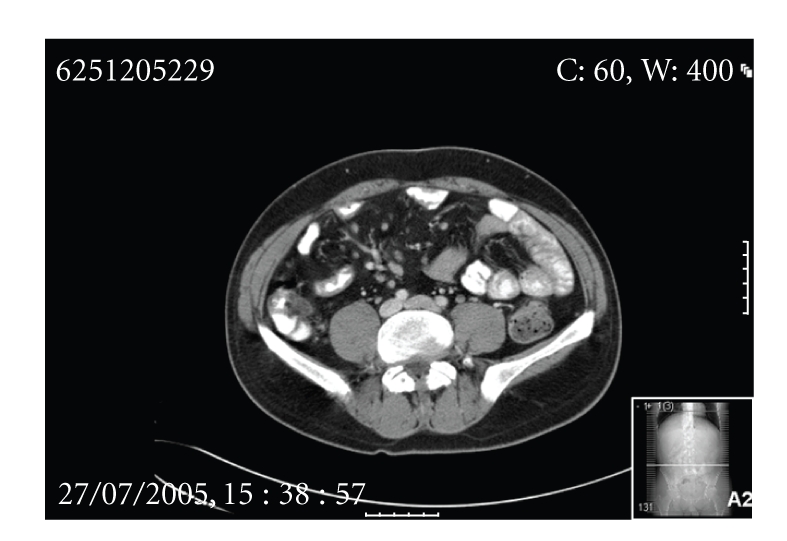
Thickening of terminal ileum and mesenteric lymphadenopathy.

**Figure 2:**
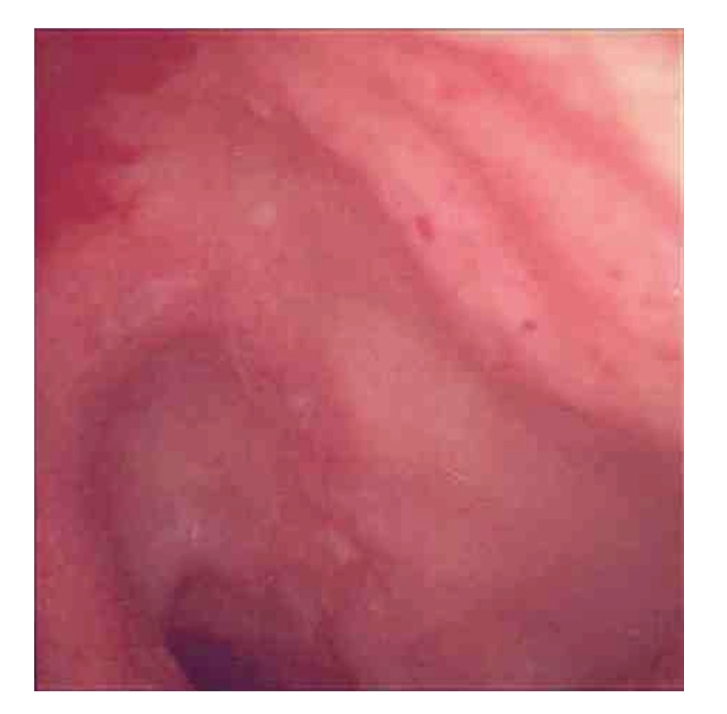
Multiple erosions in the terminal ileum seen at colonoscopy.

Five years later, a further recurrence of symptoms occurred. He was worked up in an identical fashion with colonoscopy, abdominal CT, and barium meal and follow-through. The only positive finding was on CT, which demonstrated a featureless terminal ileum, despite the wall being of normal thickness with no fat stranding. On this occasion the patient again declined diagnostic laparoscopy. Again, he responded to empirical antituberculous therapy, although a laparoscopy was not performed. A further three years later, he again presented with a recurrence of his symptoms, CT showed thickening of the terminal ileum (Figure 1), and colonoscopy demonstrated multiple erosions in the terminal ileum (Figure 2). Biopsies did not demonstrate granulomata but highlighted patchy acute and chronic inflammation with inflammatory cell infiltrate consistent with Crohn's disease. Prolonged microbiological culture of ileal specimens was also negative. On this occasion, a course of steroids for Crohn's disease was prescribed and, again, he made a dramatic recovery. 

## 3. Discussion

Crohn's disease is considered to be a disease of unknown aetiology, which is increasing in incidence in developed countries. This case report provides anecdotal evidence that supports a link between mycobacteria and Crohn's disease. Over a period of ten years, KG presented intermittently with fatigue, diarrhoea, abdominal pain, and weight loss. On the fourth episode, he was diagnosed with Crohn's disease after suggestive features on endoscopy, histology, and CT, leading to a course of steroids and full recovery. However KG went into remission on three previous episodes, for one, five, and three years, respectively, after a course of antituberculous therapy (rifampicin, isoniazid, ethambutol, and pyrazinamide). This adds weight to a body of evidence that postulates mycobacteria as an aetiological agent in Crohn's disease.

Dalziel described a chronic, interstitial enteritis two decades before the report by Crohn and colleagues [6]. He also noted the similarities between Crohn's disease and Johne's disease in cattle. Johne's disease is characterised by granuloma formation, transmural inflammation, and involvement of adjacent lymphoid tissue [7]. MAP fulfils Koch's postulates as the causative organism, since it has been identified in diseased cattle, cultured, and shown to reinfect healthy animals. MAP has also been shown to cause ileocolitis in primates [8].

However, the evidence that MAP causes intestinal disease in humans is inconsistent. Case reports of immunosuppressed patients and children have demonstrated diffuse MAP infection in humans [9, 10]. Indeed, a six-year-old boy developed typical ileocaecal Crohn's disease, five years after treatment for MAP lymphadenitis [7]. Despite these cases, efforts to identify organisms in histological specimens from patients with Crohn's disease have been disappointing. This is in contrast to Johne's disease, where the organism may occasionally be seen. A possible reason for this is the fastidious nature of the organism, which is difficult to grow in vitro [11]. Unlike M. Tuberculosis, MAP may be present in its cell-wall deficient form in humans. In this sense, the disease may resemble paucibacillary leprosy, rather than tuberculosis. In cell-wall deficient states, typical staining for acid and alcohol fast bacilli is likely to be negative. Other laboratory techniques, identifying DNA-  , RNA-  , or MAP-specific proteins, show significant interlaboratory variation which may represent environmental contamination or technical difficulties. Bernstein and colleagues have reported an increased seroprevalence of MAP in Crohn's disease measured by ELISA [12], although this finding has not been replicated with different MAP antigens. 

Several investigators have studied the effects of antimycobacterial therapy in CD. Initial, open-label studies of antimycobacterial therapy in CD suggested a possible therapeutic benefit. In 1994, Mor and colleagues [13] showed that the most effective regimen for the treatment of M. avium infection is a combination of macrolide antibiotics and rifabutin. In 1997, Gui et al. [14] described the results of treatment of Crohn's disease with clarithromycin or azithromycin and rifabutin. They called their treatment RMAT (rifabutin and macrolide antibiotic therapy). Of the 46 patients who tolerated RMAT for 6–35 months, 43 (94%) went into clinical remission. This was associated with a reduction in the Crohn's disease activity index (CDAI) and inflammatory parameters. In addition, only 2 of 19 patients who were steroid dependent at the beginning of the study continued to require steroids. However, controls were not used in this study. In 1999, Douglass and colleagues [15] reported a 6-month open pilot study of 20 patients treated with rifabutin, clarithromycin, and clofazimine. Twelve (60%) of the 20 patients who were steroid dependent, refractory, or requiring surgery were in remission by 6 months. Shafran et al. [16] reported an open trial of RMAT with a probiotic in 36 patients with active Crohn's disease. Of the patients who tolerated treatment, 58% achieved a decrease in CDAI of 70 points and did not require steroids or immunosuppressants. Borody et al. [17] also treated 12 patients with rifabutin, clarithromycin and clofazimine for 6–54 months. Six of the 12 (50%) achieved complete clinical, colonoscopic, and histological remission. 

These four, small studies suggest that half of patients with active Crohn's disease, who can tolerate long-term antibiotic therapy, will achieve remission. These figures are higher than published data for steroids, immunomodulators, or the ACCENT trials for infliximab [18]. However, a recent, well-designed, randomised, and placebo-controlled trial of clarithromycin, rifabutin, and ethambutol failed to show a sustained benefit in CD [19]. Although the antibiotic regimen showed a benefit at week 16, in addition to corticosteroid therapy, this effect was lost over the 2-year trial period. However, the presence or absence of MAP in these patients was not assessed and there is therefore no evidence that MAP was cleared by treatment. Moreover, the doses of rifabutin (450 mg), clarithromycin (750 mg), and clofazimine (50 mg) in this trial are below the optimal doses for MAP treatment, and resistance may also have developed over the three-year period despite the use of three drugs.

## 4. Conclusion

In conclusion, the case presented above is an example of anecdotal experience from many physicians of the response of CD to antimycobacterial therapy. On the back of recent insights into the complex epithelial interface between the gut mucosa and intestinal microbiota [20], a causal role for MAP in patients with genetic susceptibility to microbial persistence remains a possibility to be explored. This case study demonstrated that the patient suffered relapses at least three times which were treated successfully with antimycobacterial drugs. This suggests that the patient was most likely reinfected with the microorganism. MAP after all is present in pasteurised milk and meat.
